# Correction: Profiling HPV-16–specific T cell responses reveals broad antigen reactivities in oropharyngeal cancer patients

**DOI:** 10.1084/jem.2020038910192022c

**Published:** 2022-10-25

**Authors:** Kunal H. Bhatt, Michelle A. Neller, Sriganesh Srihari, Pauline Crooks, Lea Lekieffre, Blake T. Aftab, Howard Liu, Corey Smith, Liz Kenny, Sandro Porceddu, Rajiv Khanna

Vol. 217, No. 10 | https://doi.org/10.1084/jem.20200389 | July 27, 2020

The authors regret that in the original version of Fig. S1 B ii, the x-axis labels E5-5, E5-6, E5-7, E5-8, and E5-9 were accidentally written as E5-6, E5-7, E5-8, E5-9, and E5-10. In addition, “45 h” was corrected to “4 h” in the Fig. S1 C i legend, as indicated in bold and underline. The corrected Fig. S1 and its legend are shown here. The errors appear in PDFs downloaded before October 18, 2022.

**Figure S1.**
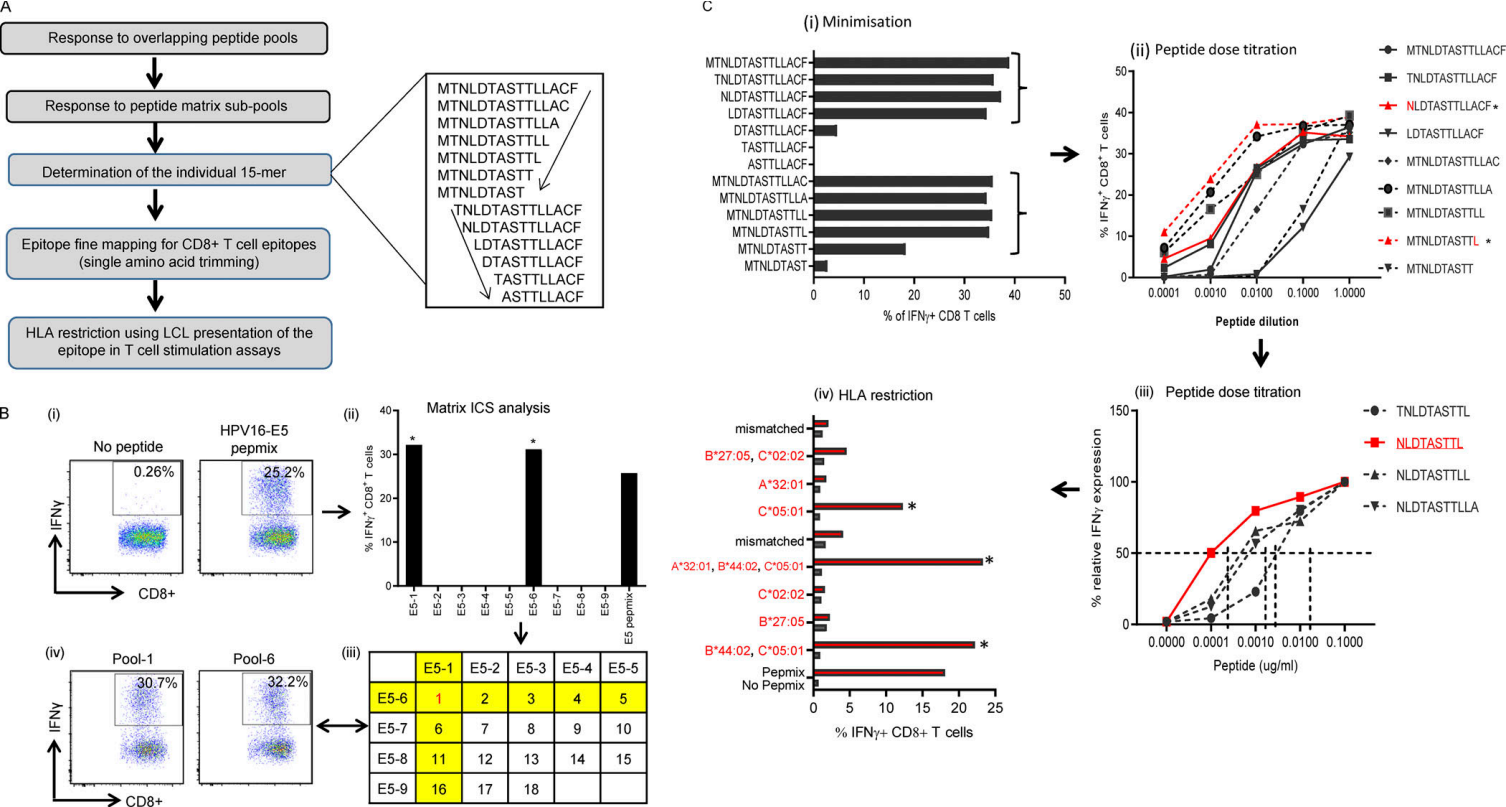
Mapping of HPV-16 epitopes recognized by CD8^+^ T cells from OPC patients. **(A)** Overview of the process for mapping HPV epitopes recognized by T cells from OPC patients. An example of the trimming of a 15–amino acid sequence to define the minimal T cell epitope is shown to the right of the flowchart. The amino acid sequence was trimmed from the C-terminal and N-terminal ends, as indicated by the arrows. **(B)** Representative data showing the identification of a T cell determinant. (i) T cell cultures were generated by stimulating PBMC with OPPs from HPV-16 antigens. After 14 d in culture, the T cells were restimulated with OPPs from individual antigens, and intracellular IFN-γ production was analyzed by flow cytometry. Reactive T cells were further analyzed to determine the cognate peptide. (ii) Subpools of peptides were made for each antigen (nine subpools for E5 are pictured) and used in an IFN-γ ICS assay. The T cell response against each subpool is shown in the bar graph. (iii) The responses against each subpool were overlaid on a two-dimensional matrix, which shows the common individual peptides among the pools. The positive responses against pools 1 and 6 are highlighted in yellow, showing the common peptide 1 in red (row and column highlighted in yellow; common peptides in red text). (iv) Flow cytometric plots showing the T cell responses against subpools 1 and 6, assessed by intracellular IFN-γ staining. **(C)** Fine epitope mapping and HLA restriction. (i) Minimization of the active epitope within the 15–amino acid sequences derived from the peptide matrix analysis. T cell cultures responding to peptide 1 from the E5 subpools were tested against a range of shorter peptides within the peptide 1 sequence. The shorter peptides were synthesized by sequentially trimming one amino acid from the N-terminus and C–terminus down to a nine–amino acid peptide. T cell cultures were assayed for the expression of IFN-γ upon stimulation with each of the trimmed peptides at a concentration of 1 µg/ml for **4 h**. The figure shows the CD8^+^ T cell response for each trimmed peptide from E5 subpool 1 (MTNLDTASTTLLACF). The peptides that stimulated a T cell response, denoted by brackets, were further analyzed in a dose titration assay. (ii) Peptide dose titration assay. T cell cultures were stimulated with serial decimal dilutions of selected minimized peptide sequences to determine the exact epitope sequence within the longer peptides. A representative example of the titration assay is shown; in this example, NLDTASTTL is the likely minimal epitope sequence. (iii) Modified peptide sequences were tested in a dose titration assay to determine if the addition of extra amino acids at the N- or C-terminus could enhance peptide specificity. The example pictured demonstrates that NLDTASTTL is the likely minimal epitope sequence targeted by HPV-16–E5–specific CD8+ T cells from this patient. (iv) Representative HLA class I restriction analysis for epitopes mapped from HPV antigens. A panel of lymphoblastoid cell lines with one HLA allele matched to the patient were loaded with each individual peptide for 1 h before coincubation with T cells for 4 h. Responses were assessed by intracellular IFN-γ staining. The OPC patients whose T cells responded to NLDTASTTL had a class I HLA type of A*32:01, B*27:05, B*44:02, C*02:02, or C*05:01. In this example, the strongest response was elicited against antigen-presenting cells expressing HLA-C*05:01; therefore, this was determined to be the HLA restriction of NLDTASTTL. Asterisk indicates positive response.

